# Changes in renin‐angiotensin‐aldosterone system during cardiac remodeling after mitral valvuloplasty in dogs

**DOI:** 10.1111/jvim.16346

**Published:** 2022-01-07

**Authors:** Chieh‐Jen Cheng, Ahmed S. Mandour, Tomohiko Yoshida, Toshihiro Watari, Ryou Tanaka, Katsuhiro Matsuura

**Affiliations:** ^1^ Laboratory of Veterinary Internal Medicine, Department of Veterinary Medicine, College of Bioresource Sciences Nihon University Fujisawa Kanagawa Japan; ^2^ Laboratory of Veterinary Surgery Tokyo University of Agriculture and Technology Fuchu Tokyo Japan; ^3^ VCA Japan Shiraishi Animal Hospital Sayama Saitama Japan; ^4^ Department of Animal Medicine (Internal Medicine), Faculty of Veterinary Medicine Suez Canal University Ismailia Egypt

**Keywords:** angiotensin II, biomarker, cardiac recovery, cardiac reverse remodeling, mitral valve repair, plasma renin activity

## Abstract

**Background:**

Information regarding changes in renin‐angiotensin‐aldosterone system (RAAS) during cardiac remodeling after mitral valvuloplasty (MVP) in dogs remains lacking.

**Hypothesis/Objectives:**

To assess the longitudinal effects of MVP on circulating RAAS activity.

**Animals:**

Eight client‐owned dogs receiving MVP for myxomatous mitral valve disease (MMVD).

**Methods:**

This is a cohort study. Plasma renin activity (PRA), angiotensin II (AT2), aldosterone (PAC), blood urea nitrogen (BUN), and creatinine concentrations, were measured in these dogs before (baseline) and at 3 consecutive monthly follow‐ups (Post‐1M, Post‐2M, Post‐3M). Echocardiography was concomitantly used to assess the process of cardiac recovery after MVP.

**Results:**

The echocardiography revealed a significant decrease in LVIDDN, LA/Ao, FS, E velocity, E/A, E′ sep, S′ lat, E′ lat, and A′ lat after MVP compared with baseline (*P* < .05). There was a significant reduction in the PRA (2.45, 3.05, 2.74 vs 8.8 ng/mL/h; *P* = .002), AT2 (466, 315, 235 vs 1200 pg/mL; *P* = .009), and PAC (39.88, 47, 54.62 vs 179.5 pg/mL; *P* = .01), respectively at Post‐1M, Post‐2M, Post‐3M compared to the baseline. Additionally, BUN and creatinine concentrations decreased from Post‐1M. The RAAS variables showed significant, weak to moderate, relationship with selected echocardiographic variables.

**Conclusions and Clinical Importance:**

Mitral valvuloplasty contributes to decreased RAAS activity in MMVD dogs, which paralleled the process of cardiac reverse remodeling up to Post‐3M. This information facilitates formulating strategies to optimize clinical outcomes for dogs after MVP.

**Abbreviations**
AT2: angiotensin II
BUN: blood urea nitrogen
CHF: congestive heart failure
LA/Ao: ratio of the left atrial dimension to the aortic annulus dimension
LVIDDN: normalized left ventricular internal dimension in diastole
MMVD: myxomatous mitral valve disease
MVP: mitral valvuloplasty
PAC: plasma aldosterone concentration
PRA: plasma renin activity
RAAS: renin‐angiotensin‐aldosterone system

## 1 INTRODUCTION

Chronic activation of the renin‐angiotensin‐aldosterone system (RAAS) plays an important role in the pathogenesis of congestive heart failure (CHF) both in human and veterinary patients. One aspect of RAAS activation in this setting is evoking potent peripheral vasoconstriction and sodium/water retention, contributing to ventricular hemodynamic loading and subsequent development of left ventricular and left atrial enlargement.^1^

Myxomatous mitral valve disease (MMVD) remains the most common acquired heart disease in dogs^2^ and the leading cause of clinically encountered CHF in dogs.^3^ There is upregulation of RAAS in dogs with cardiac remodeling secondary to MMVD, especially in those with CHF.^4^ Increasing evidence has demonstrated that mitral valvuloplasty (MVP) provides promising long‐term survival benefits in dogs with MMVD,^5^, ^6^ as expected from favorable postoperative outcomes observed in human counterparts.^7^

Recently, a study demonstrated greater RAAS inhibition in American College of Veterinary Internal Medicine (ACVIM) stage D vs stage C, which revealed the effectiveness of RAAS suppressive treatments in dogs with refractory CHF.^8^ In human, pharmacological suppression of RAAS might confer further survival benefits via promoting cardiac recovery after cardiac surgery.^9^ While ventricular unloading and subsequent reverse remodeling of the heart after the surgical restoration of mitral competence is both anticipated and documented,^10^, ^11^, ^12^, ^13^ information regarding changes in RAAS profile along the course of reverse remodeling after surgical valvuloplasty remains lacking in the veterinary literature.

In this study, we assessed the effect of surgical unloading intervention on RAAS activity by measuring plasma renin activity, angiotensin II and aldosterone concentrations in dogs before and at monthly follow‐ups after MVP. During this midterm postoperative period, alterations in other circulatory markers and echocardiographic variables considered relevant to the cardiac reverse remodeling were also documented and compared with that of the RAAS activity. We hypothesize that decline in RAAS activity will be observed along with the cardiac reverse remodeling process after mitral valvuloplasty in dogs.

## 2 MATERIAL AND METHODS

### 2.1 Animals

Client‐owned dogs receiving MVP exclusively for MMVD from August 2019 to April 2020 at a private clinic were enrolled consecutively for this study. Baseline values were obtained within 1 week before the scheduled date of surgery. Postoperatively, all dogs have at least 3 monthly follow‐up evaluations at equal intervals. Clinical diagnosis, staging, and decision for the surgical indication of MMVD were determined through comprehensive evaluation including medical history recoding, physical examination, hematology, biochemistry, diagnostic electrocardiography, radiography, and echocardiography. Specific objects/variables to be evaluated before surgery were as follows: signalment (age, breed, sex, body weight), current cardiac medication, presence of cachexia, renal function (blood urea nitrogen [BUN], creatinine), and echocardiographic variables reflecting size and function as delineated in the Echocardiography section below. Inclusion criteria included a diagnosed MMVD, an ACVIM stage of at least B2, and being surgically treated by MVP. The sole criterion for exclusion was a lack of 2 or more measurements during the follow‐up period. Before enrollment, informed client consent was obtained for every dog.

### 2.2 Mitral valvuloplasty

Mitral valvuloplasty was performed similarly to an established technique described for small dogs.^6^ In brief, the heart was approached through left intercostal thoracotomy and pericardiectomy. After left atriotomy, repair of the diseased mitral apparatus with synthetic chordal replacement followed by annuloplasty was performed under cardiopulmonary bypass.

### 2.3 Echocardiography

The echocardiographic assessment was carried out while the dog was maintained in lateral recumbency and examined under standard conditions using a sector probe of 5 MHz (Aplio 300, Canon medical system, Tokyo, Japan). Three consecutive heartbeats were recorded at the end of the expiratory phase. To evaluate structural and functional changes during the course of reverse remodeling of the left atrium and the left ventricle, combined conventional echocardiography protocol including 2‐dimensional, M‐mode, Doppler blood flow, and tissue Doppler imaging techniques from the right and left parasternal long‐ as well as short‐axes views, were obtained.^14^ Using specified view and modality, the following variables were measured at each time point. All measurements were made by 2‐dimensional echocardiography unless mentioned otherwise. On right parasternal short‐axis view, left ventricular internal dimension in diastole (LVIDd) was determined at the papillary muscle level by 2‐dimensional echocardiography, after which normalized left ventricular internal dimension in diastole (LVIDDN) was calculated from the LVIDd and body weight measured at the same time by an established allometric formula.^15^ Ratio of the left atrial dimension to the aortic annulus dimension (LA/Ao) was measured at the basal level by 2‐dimensional echocardiography. Fractional shortening (FS) was obtained at the papillary muscle level by M‐mode modality. On left parasternal long axis 4‐chamber view, early diastolic mitral inflow (E) velocity and ratio of peak velocity of early diastolic transmitral flow to peak velocity of late diastolic transmitral flow (E/A) were obtained by Doppler interrogation. Systolic (S′), early diastolic (E′), and late diastolic (A′) wave signals at septum (sep) and lateral (lat) mitral annulus, respectively were measured by tissue Doppler imaging.

### 2.4 Biochemical assessment of renin, angiotensin II, aldosterone, and renal functions

Plasma renin activity (PRA), angiotensin II (AT2), and aldosterone (PAC) concentrations were determined by radioimmunoassay (SRL Inc, Tokyo, Japan) at each time interval. For this purpose, whole blood samples were withdrawn using disposable EDTA‐treated vacutainers (Venoject II, Terumo, Tokyo, Japan). Clear nonhemolyzed plasma samples were collected after the spinning of blood at 3000 rpm for 10 minutes, frozenly stored, and the temperature strictly kept at −20°C. To minimize the postsampling ex vivo effect on the targeted RAAS activity, all frozen‐stored samples were sent on dry ice within 1 week after collection for measurement, where pretreatment of assay‐specific inhibitors was conducted in accordance with the manufacturer's instructions. The timing of sampling depended on the client's convenience for the visit. Feeding time and diet content were variable among the dogs. For other clinical biochemical tests including measurement of plasma BUN and creatinine, heparinized plasma samples were utilized immediately after centrifugation (DRI‐CHEM 7000, FUJIFILM, Tokyo, Japan).

### 2.5 Statistical analysis

Statistical differences between groups were determined by repeated 1‐way analysis of variance for nonparametric data using Friedman test. Post hoc testing was done using Dunn's Multiple Comparison Test. A *P*‐value less than .05 was considered to indicate the Gaussian approximation of nonparametric data. Nonparametric data are presented as median and range. All analyses were conducted using GraphPad Prism 8.0 (GraphPad Software, San Diego, California). Spearman's rank correlation and linear regression analysis were used to assess the relationship between RAAS and conventional echocardiographic variables. Coefficient of determination (*R* ^2^) was computed from the sum of the squares of the distances of the points from the best‐fit curve.

## 3 RESULTS

### 3.1 Study sample

A total of 8 dogs met the inclusion criteria and were enrolled in this study. None were excluded during the follow‐up period. Breeds included Chihuahua (n = 3), Cavalier King Charles spaniel (n = 1), Chin (=1), Mongrel (n = 1), Pomeranian (n = 1), and Shih Tzu (n = 1). Six dogs were male (3 intact and the other 3 neutered) while 2 were spayed female. The median [range] age and weight of the dogs were 10.5 [8–14] years and 5.025 [2.4‐7] kilogram, respectively. In accordance with the ACVIM classifications scheme, 1 dog was at stage B2 and 7 at stage C. Before surgery, 3 dogs had been prescribed furosemide, 4 had been prescribed torsemide, 7 had been prescribed pimobendane, 4 had been prescribed an angiotensin‐converting enzyme inhibitor (ACEI), 1 had been prescribed spironolactone, and 6 had been prescribed amlodipine. After surgery, all dogs were discharged within 2 weeks of postoperative care. Complete stop of RAAS‐targeting and other medications affecting hemodynamics (diuretics, inotropes, etc) after surgery was achieved at the time of discharge in all cases. Baseline characteristics and individual dosage regime of the study cohort are summarized in Table S1.

### 3.2 Echocardiographic assessment

**FIGURE 1:**
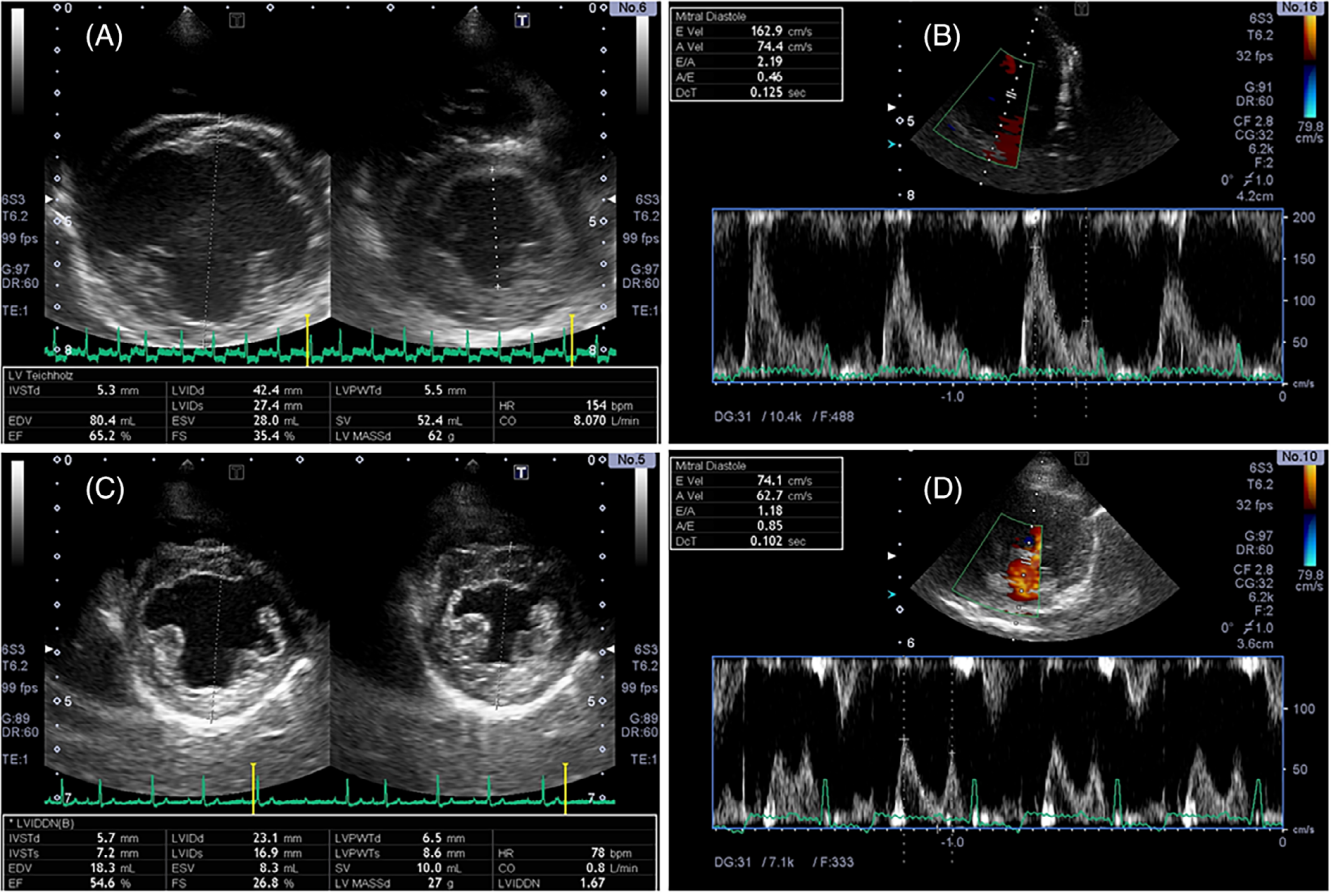
Echocardiograms of a representative case before (A and B) and 3 months after (C and D) mitral valvuloplasty demonstrating substantial cardiac reverse remodeling. Before surgery, severe eccentric left ventricular hypertrophy with an LVIDDN of 2.33 (not shown in the image) (A) from right parasternal short‐axis view, and pulse‐wave Doppler measurement from left apical 4‐chamber view demonstrating a severely elevated early mitral inflow velocity (E Vel) of 162.9 m/s (B) were presented. Three months after surgery, excellent cardiac recovery allowed significantly reduced left ventricular dimension (LVIDDN, 1.67) (C) and normalization of early mitral inflow velocity reading (74.1 cm/s) (D)

Echocardiographic data were summarized in Table 1 and Figure 1. In general, improvement in cardiac anatomical and functional characteristics was observed throughout the postoperative period compared with preoperative values, as reflected by a significant reduction in LVIDDN, LA/Ao, FS, E velocity, E/A, E′ sep, S′ lat, E′ lat, and A′ lat velocities at Post‐2M, Post‐3M compared with preoperative corresponding values (*P* < .05). Moreover, LA/Ao, FS, and S′ lat were significantly reduced at Post‐1M compared with baseline (*P* < .05). Systolic LV septal and lateral velocities (S′ sep and S′ lat) were significantly decreased at Post‐3M compared with baseline (*P* < .001, *P* < .05, respectively); meanwhile, no statistically significant change in A′ sep was observed. Collectively, these findings indicated general morphological and functional improvement of the heart after MVP.

**TABLE 1:** Conventional echocardiographic variables before (Baseline) and after mitral valvuloplasty at 3 time intervals (postoperative‐1 month, 2 month, 3 month) in the 8 dogs

Parameters	Unit	Baseline	Postoperative			*P* value
			1 month	2 month	3 month	
BW	kg	5.05 (2.5‐8.6)	4.82 (2.66‐8.05)	4.87 (2.58‐8.3)	4.75 (2.74‐8.32)	.08
LVIDd	mm	34.5 (26.7‐42.2)	26.5 (19.4‐34.4)	25.7 (16.1‐36.8)*	22.6 (17.4‐34.2)***	<.001
LVIDDN		2.13 (1.76‐2.33	1.64 (1.45‐1.96)	1.62 (1.21‐1.97)*	1.45 (1.22‐1.83)***	<.001
LA/Ao		2.21(1.92‐2.51)	1.38 (1.19‐1.77)*	1.25 (1‐1.46)***	1.39 (1‐1.71)	<.001
FS (%)	%	49.7 (35.4‐59.2)	33.4 (19.6‐55)*	36.5 (23.5‐50.2)*	34.3 (20.1‐52.9)*	.006
E velocity	cm/s	121.7(107‐164)	82.8 (53‐123)	81.8 (49‐99.5)**	74.7 (60.6‐90.2)*	.003
E/A		1.3 (0.95‐2.88)	0.66 (0.44‐1.05)	0.64 (0.38‐1.18)*	0.78 (0.57‐1.18)*	.01
S′ sep	cm/s	7.9 (5.1‐12.8)	6.30 (4.2‐8.6)	5.60 (4.7‐9)	4.42 (3.6‐6.3)**^,^ ^a^	.003
E′ sep	cm/s	6.3 (5.1‐11.9)	4.7 (3.2‐7.8)	4.2 (2.7‐6.8)*	3.6 (2.3‐9.4)**	.002
A′ sep	cm/s	5.90 (2.6‐9.9)	5.40 (3.5‐8.2)	4.55 (2.9‐8)	4.85 (2.4‐6.2)	.1
S′ lat	cm/s	10.15 (7.1‐12.2)	5.95 (3.6‐8.1)*	5.25 (4.4‐7.7)	4.73 (3.4‐6.5)***	<.001
E′ lat	cm/s	8.1 (7–10)	5.3 (3.2‐8.2)	5.0 (3.2‐9)	4.2 (2.7‐5.9)**	.009
A′ lat	cm/s	8.2 (4.5‐11.9)	5.75 (2.9‐8.5)	6.25 (3.5‐7.8)	4.55 (3.2‐5.5)*	.03

*Note*: Data are expressed as median and range. Asterisk (*) is used to compare the significance between values of baseline and postoperative observations (*, **, *** indicates *P* < .05, .01, .001, respectively).

Abbreviations: A′, late diastolic wave signal as measured by Tissue Doppler imaging; BW, body weight; E velocity, early diastolic mitral inflow velocity; E′, early diastolic wave signal as measured by Tissue Doppler imaging; E/A, the ratio of peak velocity of early diastolic transmitral flow to peak velocity of late diastolic transmitral flow; FS, fractional shortening; LA/Ao, the ratio of the left atrial dimension to the aortic annulus dimension; lat, mitral annulus at the left ventricular lateral wall; LVIDd, left ventricular internal dimension in diastole; LVIDDN, normalized left ventricular internal dimension in diastole; S′, systolic wave signal as measured by Tissue Doppler imaging; sep, mitral annulus at the septal wall.

^a^ Compares postoperative‐1 month value with that of postoperative‐2 month and postoperative‐3 month (*P* < .05). No significant difference was observed between postoperative‐2 month and postoperative‐3 month.

### 3.3 Changes in the renin‐angiotensin‐aldosterone system throughout the investigation period

**FIGURE 2:**
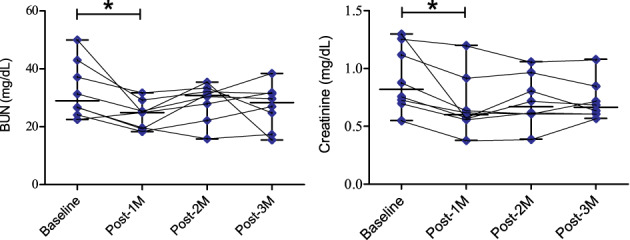
Plasma blood urea nitrogen (BUN) and creatinine concentrations before (baseline) and after mitral valvuloplasty at 3 time intervals (Post‐1M, Post‐2M, Post‐3M) in the dog study group (n = 8). Graphing with the connection of interrelated time points for each dog. Asterisk (*) indicates significance at *P* < .05

**FIGURE 3:**
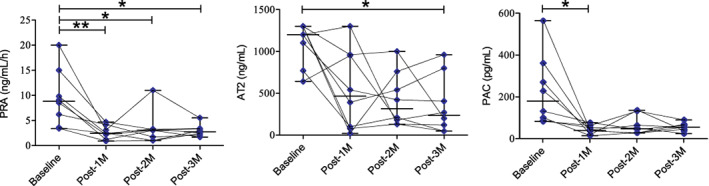
Circulatory levels of RAAS components before (baseline) and after mitral valvuloplasty at 3 time intervals (Post‐1M, Post‐2M, Post‐3M) in the dog study group (n = 8). Graphing with the connection of interrelated time points for each dog. The number of asterisks corresponds to the level of significance. *, *P* < .05; **, *P* < .01. AT2, angiotensin II; PAC, plasma aldosterone concentration; PRA, plasma renin activity

Plasma concentrations of BUN, creatinine and RAAS markers are summarized in Figure 2 and Figure 3. The concentrations of BUN and creatinine displayed a significant decrement at Post‐1M after MVP compared with the baseline values (*P* < .05; Figure 2). Besides, the activity of RAAS components were reduced after MVP. Comparing with the baseline (median [range], 8.8 [3.4‐20] ng/mL/h), PRA showed a significant reduction at Post‐1M (2.45 [0.9‐4.7] ng/mL/h; *P* < .01), Post‐2M (3.05 [1–11] ng/mL/h; *P* < .05), and Post‐3M (2.74 [1.7‐5.5] ng/mL/h; *P* < .05; left hand side of Figure 3). The concentration of AT2 was 466.1 [18‐1300] pg/mL and 315.7 [130‐1000] pg/mL at Post‐1M and Post‐2M, respectively (*P* > .05), and significantly reduced at Post‐3M (235 [46.4‐960] pg/mL) compared with the baseline (1200 [640‐1300] pg/mL; *P* < .01; center of Figure 3). Plasma aldosterone concentration showed a significant reduction at Post‐1M (39.88 [14.3‐76.7] pg/mL) compared with the baseline (179.5 [82.9‐564] pg/mL; *P* < .05; right hand side of Figure 3). The median values of the PRA, AT2, and PAC are summarized, in chronological order, as follows: 8.8, 2.45, 3.05, 2.74 ng/mL/h; 1200, 466, 315, 235 pg/mL, and 179.5, 39.88, 47, 54.62 pg/mL, respectively. No significant difference between any postoperative time intervals were detected for all RAAS variables (*P* > .05).

### 3.4 Correlation between RAAS and cardiac measurements

Table 2 summarizes the correlation results between RAAS concentrations and obtained echocardiographic variables. Plasma renin activity showed a significant positive correlation with LVIDDN, FS, E velocity, E/A ratio, S′ lat, A′ sep, E′ lat, and A′ lat (*r*_s_ = .37, .47, .42, .47, .46, .36, .36, .62; *P* < .05), respectively. Plasma AT2 concentration displayed significant positive correlation with LVIDDN, E′ sep, A′ sep, E′ lat, and A′ lat (*r*_s_ = .36, .43, .49, .35, .51; *P* < .05), respectively. In addition, PAC exhibited significant positive correlation with E/A ratio and S′ sep (*r*_s_ = .36, .58; *P* < .05), respectively.

**TABLE 2:** Correlation analysis between RAAS levels and echocardiographic variables

Parameters	PRA		AT2		PAC	
	*r*_s_	*P* value	*r*_s_	*P* value	*r*_s_	*P* value
LVIDDN	.371*	.04	.355*	.05	.333	.06
LA/Ao	.273	.13	.281	.12	.229	.21
FS	.466**	.007	.231	.2	.242	.18
E	.42*	.02	.027	.89	.266	.14
E/A	.465**	.007	.179	.33	.359*	**.04**
S′ sep	.458**	.008	.133	.47	.576**	**.001**
E′ sep	.296	.1	.426*	.02	.086	.64
A′ sep	.533**	.002	.487**	.005	.143	.43
S′ lat	−.041	.82	.008	.97	−.061	.74
E′ lat	.36*	.04	.347*	.05	.174	.34
A′ lat	.623***	.00	.513**	.003	.172	.35

*Note*: Spearman's correlation between PRA, AT2, and PAC and echocardiographic measurements in the canine study group. *, **, *** respectively represent significance *P* < .05, *P* < .001, *P* < .0001 and are shown in bold (n = 8).

Abbreviations: A′, late diastolic wave signal as measured by Tissue Doppler imaging; AT2, angiotensin II; E, early diastolic mitral inflow velocity; E′, early diastolic wave signal as measured by Tissue Doppler imaging; E/A, the ratio of peak velocity of early diastolic transmitral flow to peak velocity of late diastolic transmitral flow; FS, fractional shortening; LA/Ao, the ratio of the left atrial dimension to the aortic annulus dimension; lat, mitral annulus at the left ventricular lateral wall; LVIDd, left ventricular internal dimension in diastole; LVIDDN, normalized left ventricular internal dimension in diastole; S′, systolic wave signal as measured by Tissue Doppler imaging; sep, mitral annulus at the septal wall; PAC, plasma aldosterone concentration; PRA, plasma renin activity.

### 3.5 Effect of RAAS on cardiac measurements

Coefficient of determination (*R* ^2^) obtained from linear regression analysis between RAAS and cardiac measurements was illustrated in Table 3. There was a significant effect of PRA on LVIDDN, LA/Ao, FS, E velocity, E/A ratio, S′ lat, and E′ lat (*R* ^2^ = .15, .32, .19, .43, .43, .18, .27; *P* < .05), respectively. Angiotensin II exhibited significant effect on LVIDDN, LA/Ao, S′ sep, E′ sep, S′ lat and E′ lat (*R* ^2^ = .14, .13, .20, .28, .15, .27; *P* < .05), respectively. Plasma aldosterone concentration demonstrated significant effect on LA/Ao, FS, E velocity, E/A ratio, and S′ lat (*R* ^2^ = .15, .32, .19, .43, .43, .17, .27; *P* < .05), respectively.

**TABLE 3:** Effect of RAAS levels on echocardiographic variables

Variables	PRA		AT2		PAC	
	*R* ^2^	*P* value	*R* ^2^	*P* value	*R* ^2^	*P* value
LVIDDN	.152*	.03	.135*	.04	.109	.07
LA/Ao	.323**	.001	.129*	.04	.143*	.03
FS	.191*	.01	.004	.72	.239**	.005
E velocity	.426***	<.001	.096	.09	.196*	.01
E/A	.427***	<.001	.092	.09	.323**	.001
S′ sep	.034	.31	.2*	.01	.025	.39
E′ sep	.108	.07	.279**	.002	.082	.11
A′ sep	.023	.41	.007	.65	.000	.94
S′ lat	.178*	.07	.15*	.03	.142*	.03
E′ lat	.274**	.002	.269**	.002	.014	.51
A′ lat	.028	.36	.059	.18	.002	.8

*Note*: Coefficient of determination (*R* ^2^) obtained from linear regression analysis of systemic renin‐angiotensin II‐aldosterone levels (PRA, AT2, PAC) and echocardiographic measurements in the canine study group (n = 8). *, **, *** respectively represent significance *P* < .05, *P* < .001, *P* < .0001 and are shown in bold.

Abbreviations: A′, late diastolic wave signal as measured by Tissue Doppler imaging; AT2, angiotensin II; E velocity, early diastolic mitral inflow velocity; E′, early diastolic wave signal as measured by Tissue Doppler imaging; E/A, the ratio of peak velocity of early diastolic transmitral flow to peak velocity of late diastolic transmitral flow; FS, fractional shortening; LA/Ao, the ratio of the left atrial dimension to the aortic annulus dimension; lat, mitral annulus at the left ventricular lateral wall; LVIDd, left ventricular internal dimension in diastole; LVIDDN, normalized left ventricular internal dimension in diastole; S′, systolic wave signal as measured by Tissue Doppler imaging; sep, mitral annulus at the septal wall; PAC, plasma aldosterone concentration; PRA, plasma renin activity.

## 4 DISCUSSION

The current study aimedto elucidate the effect of MVP on RAAS in dogs with MMVD, the understanding of which contributes to the formulation of an adaptive strategy of RAAS modulation, thereby optimizing postsurgical myocardial recovery and thus the clinical outcome in dogs after MVP. Knowledge of whether RAAS activation persisted after MVP would be useful to determine whether RAAS suppression is needed postoperatively, which it is controversial in human medicine.^9^, ^16^, ^17^

Starting from the first month after surgery, a significant difference from the baseline presurgical value was detected in most echocardiographic and RAAS variables. The difference had lasted over the postoperative 3‐month midterm period. Our result implies that in parallel with anatomical/functional normalization of the heart (ie, evidence of cardiac reverse remodeling) there exists a corresponding decrease in the RAAS activity after the MVP. This is additionally supported by the discovery of statistically significant, though weak to moderate, correlation and linear regression relationships between the RAAS levels and echocardiographic variables. The moderate‐to‐low values of *R* ^2^ from linear regression analysis suggest that the RAAS and echocardiographic variables are only related to some degree. Other conceivable confounding factors include elimination of mitral regurgitation by the surgery per se, concomitant cessation of diuretic and other cardiac medications after surgery, or both. Specifically, the circulatory level of the 3 major components of the RAAS (renin, angiotensin II, and aldosterone) was found to decrease by the first month after MVP in most dogs, even though the extent of this reduction did not reach reference ranges inferred from several other studies involving the same measurements of healthy dogs (PRA median, 0.89‐2.5 ng/mL/h; AT2 mean, 14‐20 pg/mL; PAC median, 14.5‐61 pg/mL).^4^, ^18^, ^19^, ^20^ Comparability between these historical values and our result, however, could not be fully guaranteed as the sample handling in the current study, where the addition of the assay‐specific inhibitor was not performed until the sample arrived at the laboratory, might be distinct from those studies where inhibitors were added immediately after phlebotomy. This distinction might have contributed to the relatively elevated activities of RAAS, especially angiotensin II, in this study compared to historical ones. In another case report where a dog with preoperative acute kidney injury underwent MVP, a lowering of RAAS activity was also observed to be obvious as soon as 2 days after the surgery, at which time the case was receiving intravenous fluid therapy. This might imply that the tempering effect of valvuloplasty (together with all associated changes in management after the surgery) on RAAS is postoperatively immediate, in terms of days, and substantial enough to allow the kidney to withstand fluid loading without reevoking RAAS activation, even in the setting of a decreased renal function.^21^ On the other hand, the echocardiography result signified a more complete geometric normalization from the first postoperative month: medians of LVIDDN and LA/Ao significantly reduced respectively to <1.7 and <1.6, the 2 most reliable clinical cardiomegaly criteria indicating treatment.^2^ These results support the discontinuation of RAAS inhibitors after MVP.

In humans, there is continued tissue RAAS activation after various surgical interventions,^22^, ^23^ with some reports concluding that pharmacological blockade of RAAS after cardiovascular surgeries is prognostically beneficial.^9^, ^16^, ^17^, ^24^, ^25^, ^26^ Since the current study focused on the systemic instead of local components of RAAS, results of tissue RAAS evaluation could be different but were not evaluated. We do currently believe that no further recovery in the cardiovascular system is required for our dogs. Meanwhile, the prognostic significance of residual marginally elevated RAAS activity (medians at the third postoperative month, namely, 2.75 ng/mL/h for PRA, 235 pg/mL for AT2, and 54.62 pg/mL for PAC) in dogs after MVP warrants continual exploration.

The results of this study might also be relevant to dogs with MMVD that do not receive surgical treatment. RAAS activity at 3 months after MVP where the echocardiographic variables improved to the point that matches B1 criteria was only slightly elevated than the previously reported reference values for dogs. On the other hand, levels of echocardiographic variables and AT2 were not as low at 1 month after the surgery compared with those at 3 months. Our data is unique in that RAAS activity was shown to be associated, at least to some extent, with echocardiographic indicators that reflect the severity of the disease. From this perspective, postoperative echocardiographic changes and RAAS might as well be applicable in reverse chronological order, in other words, natural MMVD progression from mild to severe. Though indirectly, our study supports the relationship between RAAS variables and cardiac enlargement. This information adds to our knowledge about when during the preclinical phase of MMVD the neurohormonal system could become activated. This issue is of clinical significance when determining at what time administration of RAAS blockers should be started for cases and studies dealing with naturally occurring MMVD.

To conclude, the present work focused principally on the midterm outcome of MVP for dogs with MMVD, which demonstrated a modest reduction in RAAS activity and sufficient cardiac reverse remodeling such that further RAAS blockade is considered unwarranted.

Limitations of the current study include the small number of animals enrolled and consequently inability to perform further assignment to subgroups according to the stage of MMVD, heterogeneity of dogs in various characteristics (breed, body weight, medication history, diet, and MMVD stage), time lag between the blood withdrawal and actual inhibitor addition before the assay, inconsistency in the sampling circadian time, and incomprehensive and short‐term documentation of RAAS.

One potential bias is the differences in administered medication regimes, especially those affecting hemodynamics (diuretics,^27^, ^28^, ^29^, ^30^ inotropes, RAAS antagonists, etc) and therefore, directly or indirectly, impacting RAAS. Among the prescribed contents before surgery, diuretics (7/8 dogs) and amlodipine (6/8 dogs) activate the RAAS, whereas ACEIs (4/8 dogs) and spironolactone (1/8 dogs) suppress the system. Inodilator pimobendane prescribed for the 7/8 dogs added to the complexity of the presurgical RAAS status. Apart from the technical reason of sample handling, as a group, the overall high values of baseline AT2 and PAC might suggest that pharmacological RAAS suppression was inadequate, which might be because of various unverifiable reasons such as aldosterone breakthrough, underdosing relative to the RAAS‐stimulating drugs, poor owner compliance, and so forth. All these hemodynamic influencing drugs were no longer required as soon as hospital discharge after MVP. While drug cessation is for sure a substantial benefit of the surgery from the client's point of view, this act of withdrawal of both positive and negative RAAS‐impacting medications, on the other hand, complicates the evaluability of contribution by MVP per se on the improved RAAS variables. Nevertheless, given the short (in terms of hours) elimination half‐life profile of drugs administered and the dynamic nature of RAAS, the confounding effect of the discrepancy of medication content and dosage among different individuals in the study cohort on the midterm postoperative results of RAAS activity, at least, was considered minimal.

Furthermore, only conventional components of RAAS were considered in this relatively short‐term study. Recently, alternative RAAS pathways have been described and investigations regarding their interaction with pharmacological RAAS blockade^31^, ^32^ and usefulness as biomarkers/prognostic factors^33^, ^34^, ^35^ have revealed their clinical values. This, together with the fact that inadequate normalization of the RAAS was documented in this study, indicates that further long‐term investigation into the elements of alternative RAAS in dogs after MVP is warranted for a more comprehensive understanding of the reverse remodeling process.

## 5 CONCLUSIONS

The present study demonstrated midterm decrease in RAAS activity after MVP in MMVD dogs, which paralleled the process of reverse remodeling up to the third postoperative month.

## CONFLICT OF INTEREST DECLARATION

Authors declare no conflict of interest.

## OFF‐LABEL ANTIMICROBIAL DECLARATION

Authors declare no off‐label use of antimicrobials.

## INSTITUTIONAL ANIMAL CARE AND USE COMMITTEE (IACUC) OR OTHER APPROVAL DECLARATION

Approved by VCA Japan Shiraishi Animal Hospital IACUC, protocol number R2‐062.

## HUMAN ETHICS APPROVAL DECLARATION

Authors declare human ethics approval was not needed for this study.

## Supporting information


**Table S1**. Baseline characteristics and individual dosage regime of the study cohort.
