# Supplementary material for: Changes in renin‐angiotensin‐aldosterone system during cardiac remodeling after mitral valvuloplasty in dogs
Source: J Vet Intern Med. 2022 Jan 7;36(2):397–405. doi: 10.1111/jvim.16346 (PMC8965262; doi:10.1111/jvim.16346)
Supplement: Supplementary file 1 — Table S1. Baseline characteristics and individual dosage regime of the study cohort. [file JVIM-36-397-s001.pdf]

**Table S1.** Demographic data of the eight canine patients just before mitral valvuloplasty.

| Breed | Gender | Age (Year) | BW (kg) | ACVIM Stage | Cardiac Medication (mg/kg) | Day of discharge after surgery |
|---|---|---|---|---|---|---|
| Mongrel | MI | 10 | 5.6 | C | T 0.09 q24h, P 0.44 q12h, Am 0.22 q12h | 6 |
| Pomeranian | FS | 12 | 4.45 | C | F 1.1 q12h, P 0.56 q12h, Am 0.56 q24h | 6 |
| Chin | MN | 8 | 7 | B2 | P 0.36 q12h, Am 0.14 q12h | 6 |
| Chihuahua | FS | 10 | 4 | C | T 0.05 q24h, Am 0.47 q24h | 6 |
| Chihuahua | MI | 10 | 4.15 | C | T 0.1 q12h, P 0.3 q12h, B 0.3 q12h | 8 |
| Chihuahua | MI | 14 | 2.4 | C | F 1.5 q12h, P 0.5 q12h, Al 1.0 q12h, Sp 2.0 q12h, Am 0.1 q24h | 10 |
| CKCS | MN | 11 | 6.95 | C | T 0.1 q24h, P 0.45 q8h, Al 1.5 q12h, Am 0.1 q24h | 6 |
| Shih Tzu | MN | 11 | 6.8 | C | F 2.0 q12h, P 0.37 q12h, B 0.37 q12h | 12 |

Abbreviations: ACVIM, American College of Veterinary Internal Medicine; Al, alacepril; Am, amlodipine; B, benazepril; CKCS, Cavalier King Charles spaniel; F, furosemide; FS, female spayed; MI, male intact; MN, male neutered; P, pimobendan; Sp, spironolactone; T, torsemide.
